# Benign Adrenal Adenomas Are Associated With Reduced Prevalence of Hospitalised Patients With COVID‐19

**DOI:** 10.1111/cen.70055

**Published:** 2025-11-06

**Authors:** Alan Kelsall, Chris Johns, Eleanor Hills, Jenny Zhao, John Newell‐Price

**Affiliations:** ^1^ School of Medicine and Population Health University of Sheffield Sheffield UK; ^2^ Dept of Endocrinology Sheffield Teaching Hospitals NHS Foundation Trust Sheffield UK; ^3^ Dept of Radiology Sheffield Teaching Hospitals NHS Foundation Trust Sheffield UK

**Keywords:** adrenal adenoma, COVID‐19, mild autonomous cortisol secretion

## Abstract

**Introduction:**

Adrenal incidentalomas (AI) are commonly found on imaging done for indications other than to assess the adrenal glands. Prevalence increases with age and is around 10% in people over 80 years. The majority of AIs are benign adenomas, with 20%−50% exhibiting mild autonomous cortisol secretion (MACS). Clinical guidelines recommend the use of dexamethasone to improve outcomes in patients with COVID‐19 requiring oxygen.

**Hypothesis:**

Benign adrenal adenomas protect against severe COVID‐19.

**Methods:**

Reports for all computed tomography pulmonary angiogram (CTPA) scans at Sheffield Teaching Hospitals between 11 March 2020 and 10 November 2021 were assessed for details of AI. Scan requests mandated recording COVID‐19 status. Patients with a positive COVID‐19 test within 2 weeks before the CTPA were classed as COVID‐19 positive for the analyses. Duplicate scans were removed.

**Results:**

A total of 4307 CTPA scans were included. The median age was 65 (IQR 49−77) and the majority of patients were female (55.0%). Seventy‐six (1.76%) patients had a benign adenoma. COVID‐19 positivity was found in 897 (20.8%). The presence of a benign adenoma was associated with a 70% reduced odds of being COVID‐19 positive (aOR 0.30, 95% CI 0.12−0.74, *p* = 0.01), adjusting for age and sex.

**Conclusion:**

Prevalence of adrenal adenoma was associated with significantly reduced odds of being SARS‐CoV2 positive in an inpatient cohort. Secretion of mild excess cortisol (MACS) may be protective against developing severe COVID‐19.

## 1 Introduction

Adrenal incidentalomas (AI) are commonly detected on cross sectional imaging whilst investigating for pathology unrelated to the adrenal glands. With the increased use of computed tomography (CT) and magnetic resonance imaging (MRI), the detection rate of AI has increased [1]. The vast majority of AI are benign adrenal adenomas. A wide range of prevalence has been documented dependent upon the population, with between 1.05% and 8.9% found to have an adrenal adenoma in autopsy series [2]. The prevalence increases with age and is reported to be as much as 10% in people over 80 [3]. Approximately 20%−50% of these benign adrenal adenomas secrete mild excess cortisol, termed mild autonomous cortisol secretion (MACS) [3]. MACS has been associated with increased comorbidity and mortality, including hypertension, diabetes, dyslipidaemia, cardiovascular structural and functional abnormalities and malignancy [3, 4]. Susceptibility to infection has also been documented [5]. Increased mortality is observed, with cause of death due to cardiovascular disease and malignancy being reported with greater prevalence than expected in the general population [4].

Severe acute respiratory syndrome coronavirus 2 (SARS‐CoV‐2) causes a wide range of symptoms including fever, hypoxia, dyspnoea and cough [6, 7]. Studies investigating treatment options have had mixed results, but patients requiring oxygen and treated with dexamethasone had reduced 28‐day mortality and were less likely to progress and require mechanical ventilation [8, 9]. In both studies, no benefit was found in administering dexamethasone to patients who had no oxygen requirement [8, 9]. As a result guidelines recommend commencing dexamethasone in patients with COVID‐19 requiring oxygen [10].

During the COVID‐19 pandemic, we noted that of the referrals of patients with adrenal adenoma to our weekly regional endocrine multidisciplinary team (MDT) meeting, few had a history of acute COVID‐19 at the time the scan was done. This was despite the fact that CT imaging was occurring frequently throughout this period and particularly in the investigation of symptoms associated with severe COVID‐19. Therefore, we hypothesised that the prevalence of adrenal adenoma may be reduced in patients with COVID‐19 severe enough to be admitted to hospital, and that mild cortisol secretion may be affecting the natural history of COVID‐19 symptoms.

## 2 Patients and Methods

We retrospectively assessed the reports of all computed tomography pulmonary angiogram (CTPA) scans at Sheffield Teaching Hospitals (STH) NHS Foundation Trust, United Kingdom, between 11 March 2020 and 10 November 2021. CTPA was chosen as the imaging modality that was most commonly requested for patients with symptoms potentially related to severe COVID‐19, that would also allow assessment of the adrenal glands. The adrenal glands are reported on throughout the imaging series as part of the standard protocol at STH. Data relating to total inpatient admissions and total inpatient COVID‐19 diagnoses during the time period were provided by the Information Services Department, STH.

For the purpose of this study, benign adrenal adenomas were classified radiologically as being homogenous in appearance and having Hounsfield units ≤ 10, as per the European Society of Endocrinology guidelines [3].

During the pandemic, patients with symptoms related to COVID‐19 had a COVID‐19 lateral flow or polymerase chain reaction (PCR) test on admission. Occasionally, despite a negative COVID‐19 test, clinical features and chest X‐ray were in keeping with a clinical diagnosis of COVID‐19, in which case, for our analysis, the patient was treated as COVID‐19 positive.

On requesting the CTPA on Sheffield's clinical investigation system, the clinician had to designate via a mandatory box whether the patient was COVID‐19 positive or COVID‐19 negative, to avoid cross contamination and to allow the use of a dedicated COVID‐19 positive CT scanner. Patients were therefore able to be triaged into these two groups depending on results at the time of the scan.

Oxygen data was taken from the details provided at the time of the CTPA request and where not available, the patient notes were reviewed to assess for the presence or absence of supplementary oxygen.

CT report data was available in the form of a Microsoft Excel spreadsheet which contained patients' age, COVID‐19 status as entered by the user at the time of scan request, reason for request, date of request and full report of the CTPA by a clinical radiologist.

**Figure 1:**
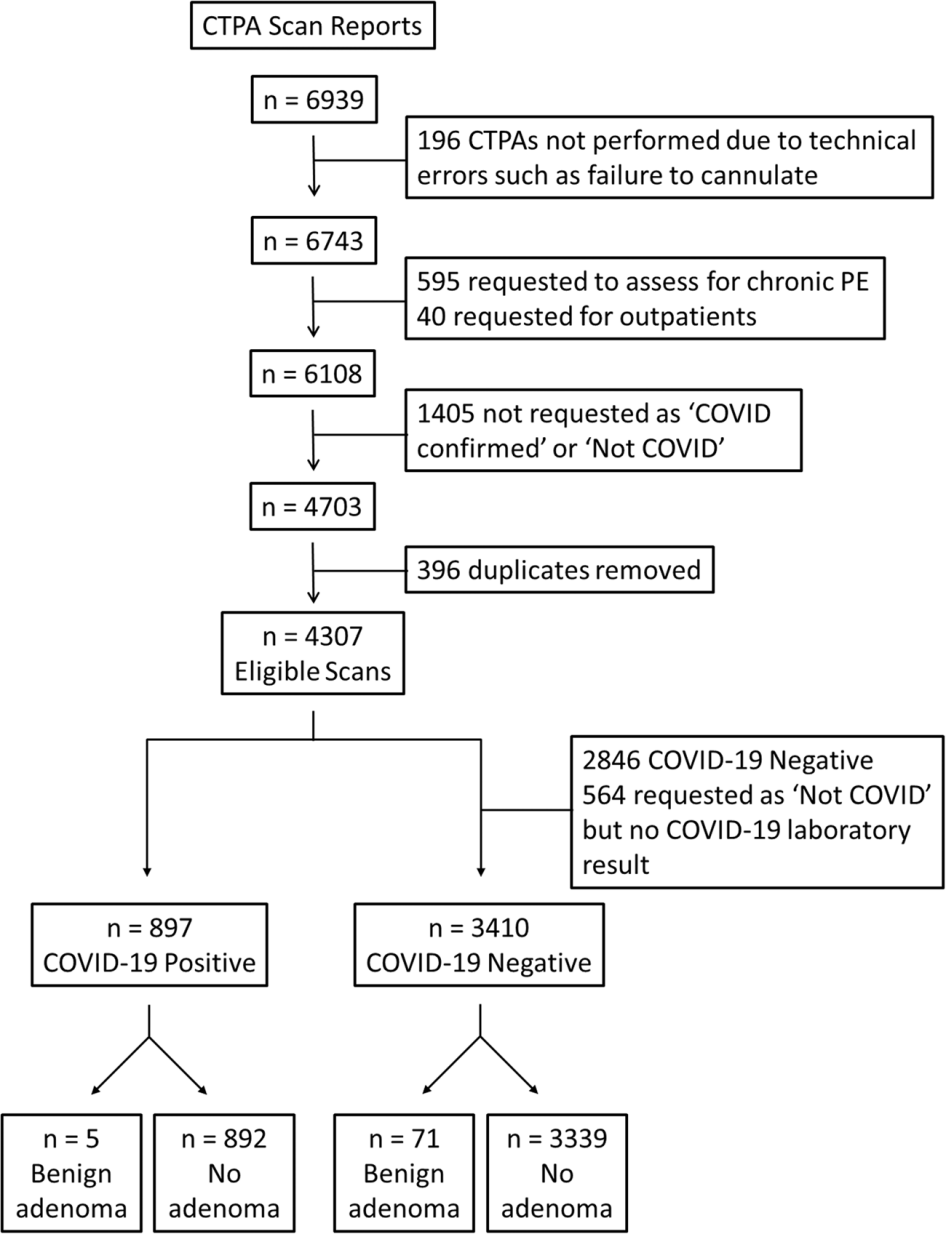
CTPA report selection flow diagram. CTPA, computed tomography pulmonary angiogram; PE, pulmonary embolism.

Image report eligibility and selection are shown in Figure 1. A number of requested scans did not occur due to technical errors such as failure to cannulate and therefore these results were removed. STH is a tertiary pulmonary hypertension centre and has a large cohort of patients with chronic pulmonary embolism (PE). CTPA imaging requested in the context of chronic PE was excluded. Requests for outpatients were removed. A small number of patients had more than one scan during the allocated time frame, and often within a short time period of each other (serial imaging). In these cases, the results from the initial scan were used and the second scan was removed.

Using Microsoft Excel filters, CTPA reports were initially screened using the search term ‘adrenal’ and reports were assessed for details of adrenal pathology. The reported COVID‐19 status at the time of CTPA request was confirmed with a documented COVID‐19 PCR or lateral flow result where possible. Patients with a positive COVID‐19 result within 2 weeks before the CTPA were classified as COVID‐19 positive for the analyses. All scans were reported by clinical radiologists at the time of the imaging. An expert radiologist re‐reviewed 10% of the scans reported as having an AI to confirm the presence of and characteristics of the AI. Mortality data is available and uploaded to the Personal Demographics Service within 1 working day. Follow‐up data for mortality was collected between September 2022 and November 2022 and simply noted as to whether the patient was alive or not at that point in time.

The study was ethically approved locally (STH19747, IRAS project ID 327729) without the need for written informed consent to analyse the imaging.

### 2.1 Statistical Analysis

All data analysis was conducted using Microsoft Excel 2010 and IBM SPSS statistics version 26. Descriptive statistics are presented as mean and standard deviation or median and interquartile range for normal and non‐normal distributions, respectively. Discrete variables were presented as frequency and percentage. Logistic regression, chi‐squared test and Fisher's exact test were performed for the analysis.

## 3 Results

A total of 4307 CTPA eligible scans were performed between 11 March 2020 and 10 November 2021 (Figure 1). Demographics are shown in Table 1. The median age was 65 (IQR 49−77) and the majority of patients were female (55.0%). An AI was reported in 124 (2.88%) patients, of which there were 76 benign adenomas. The breakdown of adrenal lesion characteristics is demonstrated in Tables 1 and 2.

**Table 1:** Demographics of all patients who had an eligible computed tomography pulmonary angiogram.

	*N*	%
Total number of patients	4307	
COVID‐19		
Positive	897	20.8
Negative	3410	79.2
Gender		
Female	2371	55.0
Male	1936	45.0
Age (median and IQR)		
All	65 (49−77)	
Female	63 (44−77)	
Male	66 (53−77)	
Adrenal incidentaloma	124	
Benign adenoma	76	61.3
Adrenocortical hyperplasia	17	13.7
Metastases	15	12.1
Indeterminate	7	5.6
Myelolipoma	4	3.2
Calcification	4	3.2
Haemorrhage	1	0.8
Mortality on follow‐up		
Alive	3200	74.3
Deceased	1107	25.7

Abbreviation: IQR, interquartile range.

**Table 2:** Further benign adrenal adenoma characteristics in patients with an eligible computed tomography pulmonary angiogram.

	*N*	%
Benign adrenal adenoma	76	
Benign adenoma site		
Left	47	61.8
Right	21	27.6
Bilateral	8	10.5
Gender		
Female	50	65.8
Male	26	34.2
Age (median, IQR)	73 (63.75−77.5)	

Abbreviation: IQR, interquartile range.

Of the 4307 CTPA scans, a total of 564 (13.1%) CTPA scans were requested as ‘not COVID‐19’ based on the patient's clinical picture, but either did not have a documented COVID‐19 test before the scan (*n* = 399) or had not had a COVID‐19 test in the previous 30 days (*n* = 165). Benign adrenal adenomas were reported in 11 CTPA scans from this group. Given that the requesting clinician considered these patients as COVID‐19 negative, they have been included in this group for analyses (Figure 1).

COVID‐19 positivity was confirmed in 897 patients (20.8%). Demographics of patients by COVID‐19 status can be seen in Table 3. There was a male preponderance in the COVID‐19 positive group (56.4%) compared with the COVID‐19 negative group (41.9%, *p* < 0.001). Median age was significantly lower in the COVID‐19 positive group (61 vs. 66, *p* < 0.001). The prevalence of benign adrenal adenoma was 0.6% in the COVID‐19 positive group compared with 2.1% in the COVID‐19 negative group.

**Table 3:** Demographics by COVID‐19 status.

	*N*	%		*N*	%	*p* value
COVID‐19 positive	897		COVID‐19 negative	3410		
Gender			Gender			
Female	391	43.6	Female	1980	58.1	
Male	506	56.4	Male	1430	41.9	< 0.001
Age (median and IQR)			Age (median and IQR)			
All	61 (47−74)		All	66 (50−78)		< 0.001
Female	58 (42−75)		Female	64 (45−78)		< 0.001
Male	63 (51−73)		Male	67 (54−78)		0.007
Adrenal incidentaloma	13	1.4	Adrenal incidentaloma	111	3.3	0.004
Benign adenoma	5	0.6	Benign adenoma	71	2.1	0.004
Adrenocortical hyperplasia	5	0.6	Adrenocortical hyperplasia	12	0.4	0.38
Unilateral	4	0.4	Unilateral	5	0.1	
Bilateral	1	0.1	Bilateral	7	0.2	
Indeterminate	1	0.1	Indeterminate	6	0.2	0.55
Metastases	1	0.1	Metastases	14	0.4	1
Myelolipoma	1	0.1	Myelolipoma	3	0.1	0.36
Calcification	0	0.0	Calcification	4	0.1	1
Haemorrhage	0	0.0	Haemorrhage	1	0.0	1
Mortality on follow‐up			Mortality on follow‐up			
Alive	672	74.9	Alive	2528	74.1	
Dead	225	25.1	Dead	882	25.9	0.63
Pulmonary embolism			Pulmonary embolism			
Acute	140	15.6	Acute	602	17.7	0.15
Chronic	0	0.0	Chronic	16	0.5	0.06
No	659	73.5	No	2613	76.6	0.049
Equivocal	98	10.9	Equivocal	179	5.2	< 0.001

Abbreviation: IQR, interquartile range.

During the period 11 March 2020 to 10 November 2021, there were 138,326 admissions to STHs and 8318 patients who had a diagnosis of COVID‐19 during their admission. Therefore, 897/8318 (10.8%) people with COVID‐19 received a CTPA scan compared to 3410/130,008 (2.6%) people without COVID‐19.

**Figure 2:**
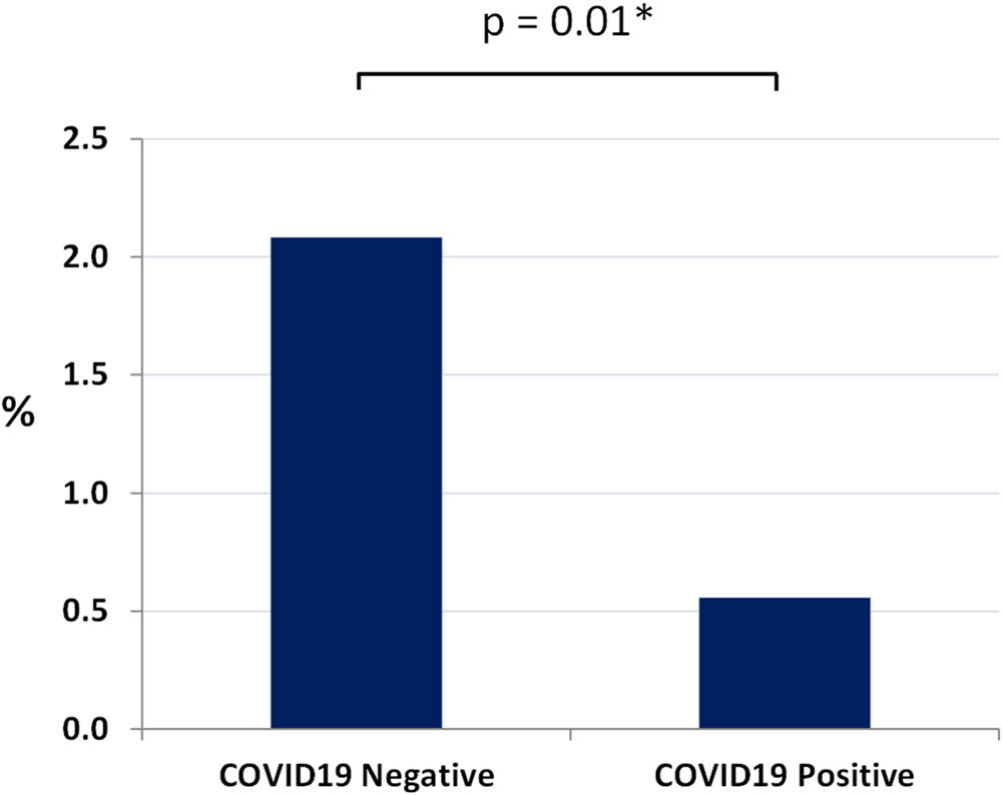
Prevalence of benign adrenal adenoma on CTPA by COVID‐19 status. *Adjusted for age and sex.

On logistic regression analysis, the presence of a benign adrenal adenoma was associated with a 74% reduced odds of the patient being COVID‐19 positive at the time of the test (OR 0.26, 95% CI 0.11−0.66, *p* = 0.004). Results were similar after adjusting for age and sex (Figure 2, aOR 0.30, 95% CI 0.12−0.74, *p* = 0.01). After excluding the cohort of 564 patients with no documented COVID‐19 test result available, the adjusted odds ratio remained the same (aOR 0.30, 95% CI 0.12−0.75, *p* = 0.01). On follow‐up, mortality (chi‐square = 0.23, *p* = 0.63) was similar between COVID‐19 groups. In addition, the presence of a benign adrenal adenoma was not associated with increased mortality at follow‐up compared to those with no adrenal adenoma (Table 4, OR 0.75, 95% CI 0.46−1.21, *p* = 0.24).

**Table 4:** Prevalence of acute pulmonary embolism and mortality data by presence of benign adrenal adenoma on computed tomography pulmonary angiogram.

	*N*	%		*N*	%	*p* value
Benign adrenal adenoma	76		No benign adrenal adenoma	4231		
Pulmonary embolism			Pulmonary embolism			
Acute	17	22.4	Acute	725	17.1	0.23
Chronic	0	0.0	Chronic	16	0.4	1
No	58	76.3	No	3214	76.0	0.94
Equivocal	1	1.3	Equivocal	276	6.5	0.09
Mortality			Mortality			
Alive	52	68.4	Alive	3148	74.4	
Deceased	24	31.6	Deceased	1083	25.6	0.24

Abbreviation: PE, pulmonary embolism.

The diagnosis of acute pulmonary embolism on CTPA was similar between COVID‐19 positive and negative groups (Table 3, 15.6% vs. 17.7%, *p* = 0.15) and also in patients with a benign adrenal adenoma on CTPA compared to those with no benign adrenal adenoma (Table 4, 22.4% vs. 17.1%, *p* = 0.23).

Upon further analysis of the COVID‐19 positive group, 719/897 (80.2%) had an oxygen requirement, as a marker of severe COVID‐19 disease. A benign adrenal adenoma was present on CTPA in 4/719 (0.6%). Compared with the COVID‐19 negative group, logistic regression analysis demonstrated a 74% reduced odds of a benign adrenal adenoma being present on CTPA in the COVID‐19 positive cohort, both unadjusted (OR = 0.26; 95% CI 0.096−0.72; *p* = 0.01) and adjusted for age and sex (aOR = 0.28; 95% CI 0.10–0.78; *p* = 0.014).

## 4 Discussion

The prevalence of AI (2.88%) and benign adrenal adenomas (1.76%) in this present study is within the range of that reported in previous autopsy and radiological studies [2]. The majority of AI were benign adrenal adenomas. Other than the difference in benign adrenal adenomas between groups, there was no statistically significant difference between other subtypes of AI in the COVID‐19 positive compared with the not COVID‐19 negative group. In addition, patients in the COVID‐19 positive group were more likely to be younger and male. AI have been reported to be more common in females on imaging studies but not on autopsy [2]. AI prevalence is well documented to increase with age [2]. To acknowledge this we adjusted for both factors in logistic regression analysis. Radiology reporter error could have affected the reporting of CTPA scans, with AI being missed, however, this would likely be the same between groups and is unlikely to be affected by the patient's COVID‐19 status.

Our results demonstrate a reduced prevalence of benign adrenal adenomas in the COVID‐19 positive group. These findings are different from those reported recently by Guclu et al. who demonstrated an increased risk in patients with adrenal adenoma (*n* = 148) of hospitalisation (30.4% vs. 21.2%) and mortality (14.7% vs. 7%) secondary to COVID‐19‐related conditions, compared to those with no adrenal adenoma (*n* = 2345) [11]. There are several reasons our findings may differ from those reported by Guclu. Firstly, the studied populations, and indeed the research question, are inherently different, with their patients presenting to a community COVID‐19 unit for assessment and only 541 (21.7%) then requiring admission to hospital compared to our study here, which focuses on the prevalence of adrenal adenoma on CTPA in those with severe COVID‐19 phenotype. In the study by Guclu et al., CT thorax was frequently done as first line to assess for COVID‐19, without requirement of a PCR test and indeed this data is missing in 37% of their total cohort. Additionally, those screened presented to a COVID‐19 unit and therefore the asymptomatic population is not captured. The adrenal adenoma group in that study were, however, both significantly older and had more comorbidities than the non‐adenoma group and on further analysis by logistic regression, the presence of an adrenal adenoma was not shown to be associated as an independent risk factor in hospitalisation (OR 1.08, 95% CI 0.67−1.75, *p* = 0.747) or mortality (OR 1.1, 95% CI 0.52−2.31, *p* = 0.808) after adjustment for comorbidities, COVID‐19 PCR status and age [11].

A limited number of studies have demonstrated an association between MACS and disruption of the immune system, with a significant increase in neutrophil to lymphocyte ratio and systemic immune‐inflammation index (calculated using neutrophils, platelets and lymphocytes as a marker of inflammation) and a reduced lymphocyte to monocyte ratio compared to nonfunctioning adrenal adenomas (NFAI) [12]. Similar findings were reported by another group who additionally noted a reduction in eosinophil count in comparison to the NFAI group [13]. A further study demonstrated an elevated IL‐6 [14]. A shift towards IL4 + T helper cell differentiation and a reduction in foxP3 + regulatory cells has also been observed in MACS compared to healthy controls, as well as upregulation of subsets of natural killer cells and reduced NKp46 expression [15].

Further detailed mapping of the immune environment was provided by Ueland et al. who found a significant increase in 46, and a decrease in three pro‐inflammatory and adverse cardiovascular biomarkers in those with MACS and Cushing's syndrome compared with healthy subjects [16]. These changes were independent of the level of cortisol excess and persisted postoperatively in patients for whom data was available. Several of the elevated cytokines in MACS, such as IL‐6, IL‐8, IL‐10, and CDCP1, are also associated with more severe disease phenotypes in COVID‐19 [17, 18], in addition to an elevated neutrophil to lymphocyte ratio [17], and this may be a reflection of HPA axis activation in severe COVID‐19. Interestingly, elevated cytokines have been associated with both mild and severe COVID‐19 with increased levels of IL‐17C, CXCL5, FGF‐21 and CCL23, elevated in MACS, found to be associated with an asymptomatic phenotype in another cohort, suggesting that immune dysregulation and length of cytokine exposure play a significant role [19].

Type 1 interferon activation is thought to play an important role in the initial innate immune response and in prevention of severe COVID‐19 [20]. Patients with genetic defects in the type 1 interferon pathway [21] or antibodies to type 1 interferons [22] had greater risk of developing severe or life threatening COVID‐19. Interferon alpha‐inducible protein 27 (IFI27) is significantly upregulated and has use as a potential biomarker for more severe infection [23, 24, 25]. The expression of IFI27 and other interferon‐stimulated genes have been shown to be downregulated following corticosteroid administration [26]. Additionally, early commencement of high‐dose dexamethasone in patients' not requiring oxygen has been reported to cause harm with increased 90 day mortality, further suggesting the importance of type 1 interferons in early phase response [27]. It is possible that in patients with MACS, where there is a continuous low‐grade secretion cortisol, that this allows the initial immune phases to occur but also modulates the response, reducing the impact of the later effects that can then be associated with the need for oxygen therapy. As such, infection would still occur but be less likely to lead to severe COVID‐19.

Conversely, it was widely observed during the pandemic that patients with obesity or type II diabetes had excess mortality from severe COVID‐19. Both of these co‐morbidities are associated with adrenal adenoma. Our observations do not fit with such an association, and suggest that any low‐grade cortisol excess is not contributing greatly to these co‐morbidities.

The strengths of the study include capturing a large number of CTPA imaging reports over a 20 month period at the height of the COVID‐19 pandemic, and the requirement to disclose COVID‐19 status at the time of CTPA request, with this confirmed by laboratory results.

This study does have limitations. Being retrospective in nature, we were unable to control for confounders. We did not have data on smoking, BMI or other comorbidities. Patients with common conditions such as diabetes were advised to shield during the COVID‐19 pandemic. As the vast majority of benign adrenal adenomas are asymptomatic, it may have been that some patients with diabetes secondary to undiagnosed adrenal adenomas shielded at home and thus COVID‐19 exposure was reduced. Additionally, MACS has been associated with increased thromboembolic events [28]. Patients with a COVID‐19 diagnosis were more likely to receive a CTPA. This may be due to the documented association with pulmonary embolism [29] and also due to the presentation of COVID‐19 patients with symptoms overlapping those of pulmonary embolism, compared to other non‐respiratory disease. All CTPA data was included within the given time period, and the reason for requesting CTPA between COVID‐19 positive and negative groups was for the assessment of pulmonary embolism, reducing confounding by scan indication, however a selection bias potentially remains given that the proportion of people who did not have a COVID‐19 diagnosis were less represented in the sample. It might be that CTPA adds an unknown bias, due to the increase in the use of this imaging modality performed during this period to assess for PE in those with COVID‐19. Moreover, during the pandemic dexamethasone suppression tests had not routinely been performed to confirm autonomous cortisol secretion from the patients with benign adrenal adenoma and our hypothesis rests on the assumption that our cohort reflects the prevalence of MACS in the general population. It is possible that the continuous, autonomous, mild excess cortisol secreted in 30%−50% of benign adrenal adenomas prevents development of severe COVID‐19. The underlying mechanism has not yet been delineated and requires further investigation.

## 5 Conclusion

In summary, our data suggest that the presence of a benign adrenal adenoma is associated with significantly reduced odds of being SARS‐CoV‐2 positive in hospitalised patients. The underlying mechanism could be associated with the mild excess cortisol secretion being protective against developing severe COVID‐19.

## Conflicts of Interest

A.K. reports a consultancy payment from HRA Pharma. J.N.‐P. reports research grants and consultancy payments to the University of Sheffield from Crinetics, Diurnal, HRA Pharma and Recordati Rare Diseases. The other authors declare no conflicts of interest.
